# LINC00657/miR-26a-5p/CKS2 ceRNA network promotes the growth of esophageal cancer cells via the MDM2/p53/Bcl2/Bax pathway

**DOI:** 10.1042/BSR20200525

**Published:** 2020-06-02

**Authors:** Xiao-Mei Zhang, Jian Wang, Zhu-Long Liu, Hong Liu, Yu-Feng Cheng, Tao Wang

**Affiliations:** 1 Department of Radiotherapy, Qilu Hospital, Cheeloo College of Medicine, Shandong University, Jinan, Shandong 250012, China; 2 Department of Ultrasound, Shandong Province Coal Taishan Sanatorium, Tai’an, Shandong 271000, China

**Keywords:** ceRNA, CKS2, esophageal cancer, LINC00657, miR-26a-5p

## Abstract

LncRNA LINC00657 has oncogenic or anti-carcinoma roles in different cancers, and yet its detailed molecular mechanism in esophageal cancer (EC) remains unclear. In addition, competitive endogenous RNA (ceRNA) regulatory lncRNA–miRNA–mRNA networks are critical for tumorigenesis and progression. Hence, the present study explored the roles of LINC00657 in EC and identified its relevant ceRNA network. We first detected the expression of LINC00657 in EC. Then, we applied starBase and TargetScan websites to find miR-26a-5p binding to LINC00657 and obtain CKS2 as a target of miR-26a-5p. The roles of LINC00657, miR-26a-5p or CKS2 in the proliferation, migration, invasion, and apoptosis of EC cells were respectively assessed by CCK-8, wound healing assay, transwell invasion assay, and flow cytometry. The changes of the MDM2/p53/Bcl2/Bax pathway were measured via Western blot. The results revealed that LINC00657 showed an aberrant high expression in EC cells, which promoted the growth of EC cells. Additionally, LINC00657 functioned as a sponge of miR-26a-5p, and LINC00657 negatively mediated miR-26a-5p to regulate the growth of EC cells. Furthermore, CKS2 was observed as a direct target of miR-26a-5p, and CKS2 controlled the growth of EC cells via the MDM2/p53/Bcl2/Bax pathway. Moreover, there was a positive correlation between LINC00657 and CKS2. LINC00657 knockdown inhibited CKS2 expression to suppress the proliferation, migration, and invasion of EC cells and induced apoptosis via regulating the MDM2/p53/Bcl2/Bax pathway. Collectively, LINC00657/miR-26a-5p/CKS2 ceRNA network could promote the progression of EC, which is good for understanding the molecular mechanism of EC and offers novel biomarkers for EC diagnosis and therapy.

## Introduction

Esophageal cancer (EC) is one of the most common cancers and the sixth leading cause of cancer death in the world [1]. Esophageal adenocarcinoma (EAC) and esophageal squamous cell carcinoma (ESCC) are known as two main histological types of EC [2]. There are approximately 79% of ESCC cases occurring in the Asian countries, while the incidence of EAC has a rapid increase in Western industrialized nations [3]. Risk factors for ESCC mainly include poor nutrition, nicotine consumption, hot beverage drinking, and alcohol abusing [4]. Barrett’s esophagus (BE), advancing age, gastric reflux, male sex, smoking, and obesity are known as EAC’s risk factors [5]. Patients with EC have poor prognosis with 15–25% of a 5-year survival rate [6]. Esophagectomy, alongside neoadjuvant chemoradiotherapy, is considered as the gold standard therapy for localized EC [7]. However, surgical resection, an invasive and complex treatment, may cause severe postoperative complications, thus leading to death [5]. In advanced or metastatic EC, surgery is generally not a suitable choice, but alternative treatments such as chemotherapy rarely improve overall survival [8]. Hence, finding new therapeutic approaches for EC is still urgent and necessary.

Long non-coding RNAs (lncRNAs, >200 nt) lack the ability to code protein [9]. Overwhelming studies suggest that lncRNAs regulate the expression level of gene through different epigenetic mechanism such as chromatin remodeling, splicing regulation, and functioning as sponges for miRNAs [10]. The aberrant expression of lncRNAs is observed in diverse cancers, which is critical for the progression of cancers [11]. LncRNA LINC00657, also named NORAD, is involved in DNA damage [12]. Previous studies indicate that LINC00657 has carcinogenic effects on different cancers mainly including colorectal cancer, lung cancer, and gastric cancer [13–15], while LINC00657 also functions as a tumor suppressor in several cancers containing glioblastoma [16]. In addition, LINC00657 overexpression in ESCC is correlated with poor prognosis [17]. Sun et al. suggest that LINC00657 promotes the growth of ESCC cells via mediating miR-615-3p [18]. However, the detailed molecular mechanism of LINC00657 in EC remains unclear.

Therefore, we first explored the effect of LINC00657 on EC. We then identified miR-26a-5p binding to LINC00657 and observed cks2 as a target of miR-26a-5p, according to ‘ceRNA hypothesis’. Several reports suggest that miR-26a-5p can inhibit the proliferation and metastasis of EC [19–21], but the more detailed mechanism of miR-26a-5p in EC still need to be further studied. CKS2 is known as a carcinogenesis factor in many cancers including EC [22,23]. Besides, miR-26a-5p is proved to effectively suppress cell proliferation and tumorigenesis through targeting CKS2 [24,25], and yet the relationship of miR-26a-5p with CKS2 in EC has not been reported. Therefore, we further studied the effect of the interaction among LINC00657, miR-26a-5p and cks2 in EC.

## Materials and methods

### Cell culture and transfection

EC cell lines (KYSE-150, ECA-109) and human normal esophageal epithelial cells (HEEC) were obtained from the American Type Culture Collection (ATCC, Manassas, U.S.A.). All cell lines were cultured in RPMI 1640 supplement with 10% FBS (fetal bovine serum) and 1% penicillin/streptomycin, maintaining in a humidified incubator at 37°C with 5% CO_2_.

MiR-26a-5p mimic, miR-26a-5p inhibitor, LINC00657 siRNA, LINC00657 plasmid, cks2 siRNA, cks2 plasmid, and the corresponding control vectors were purchased from Invitrogen (Carlsbad, CA). We applied the vector to transfect into the cells using Lipofectamine 3000 (Invitrogen).

### Quantitative reverse transcription (qRT)-PCR

Total RNA from EC cell lines (KYSE-150, ECA-109) were extracted with TRIzol reagent (Thermo Fisher Scientific). For lncRNA and mRNA, PrimeScript RT reagent Kit (TaKaRa, Japan) was applied to reversely transcribe RNA into cDNA. For miRNA, we conducted reverse transcription using the TaqMan microRNA Reverse Transcription Kit (Applied Biosystems). Then, real-time PCR was carried out under an ABI 7500 Fast Real-Time PCR system using a SYBR® Premix Ex Taq™ RT-PCR Kit (Takara). The relative gene level was assessed by the 2^−ΔΔCq^ method. The sequence of the primers was shown in Table 1. β-Actin and U6 were respectively used as endogenous controls for the normalization of lncRNA, mRNA, and miRNA.

**Table 1:** The sequence of the primers

Name	Sequence (5′-3′)
LINC00657 forward	AGCGAAGTCCCGAACGACGA
LINC00657 reverse	TGGGCATTTCCAACGGGCCAA
miR-26a-5p forward	UCCAUAAAGUAGGAAACACUACA
miR-26a-5p reverse	CAGUACUUUUGUGUAGUACAA
cks2 forward	TTCGACGAACACTACGAGTACC
cks2 reverse	GGACACCAAGTCTCCTCCAC
β-Actin forward	AGCCTCGCCTTTGCCGA
β-Actin reverse	CTGGTGCCTGGGGCG
U6 forward	CTCGCTTCGGCAGCACATATACT
U6 reverse	ACGCTTCACGAATTTGCGTGTC

### Cell counting kit-8 (CCK-8) assay

The transfected cells were seeded and incubated in 96-well plates (1 × 10^4^ cells per well) for 1, 2, and 3 days. At indicated time, CCK-8 solution (10 μl) was added into the cells, followed by 2 h of incubation. Subsequently, the absorbance of the cells at 450 nm was examined via a microplate reader.

### Wound healing assay

After transfection, the cells were added into 6-well plates followed by incubating to 100% confluence in RPMI 1640 medium with 10% FBS. Next, we used 200 μl pipette tip to scratch a vertically lineation on the plate, followed by removing the cell debris using PBS. Subsequently, the cells were cultured for another 24 h in RPMI 1640 medium without serum. The scratch was photographed under a light microscope (Olympus Corporation) at two different time points (0 and 24 h).

### Transwell invasion assay

After transfection, EC cells were harvested, resuspended in RPMI 1640 medium without serum, and seeded in the upper chamber of the 24-well transwell (Corning; pore size 8 μm) pre-coated with Matrigel (BD Biosciences). Meanwhile, we added RPMI 1640 medium with 10% FBS into the lower chamber. After 24 h of the cultivation, the cells stayed in the upper chamber were discarded with cotton swabs, while the cells invaded in the lower surface were fixed using 4% paraformaldehyde for 30 min, stained for 20 min with 0.1% Crystal Violet, and washed using PBS. These cells were recorded by a light microscope (Olympus Corporation).

### Flow cytometry

After transfection for 48 h, the cells were harvested and resuspended in binding solution (1 × 10^6^ cells/ml). Next, we added Annexin V-FITC/PI into the cells for 15 min incubation at room temperature in the dark. Then, the samples were assessed by a BD Biosciences Accuri C6 flow cytometer (Rantai Biotech, Minhang, Shanghai, China).

### Dual luciferase reporter assay

The miR-26a-5p wild‐type (WT) or mutant (MUT) putative binding site in 3′-UTR of LINC00657 or cks2 was constructed and cloned into the psiCHECK–2 vector (Promega Corporation). miR-26a-5p mimics or mimics control with LINC00657-WT, LINC00657-MUT, cks2-WT, or cks2-MUT were co-transfected into EC cell lines using Lipofectamine 3000 (Invitrogen), followed by 48 h of incubation at 37°C. Then, the luciferase activity was detected by the Dual Luciferase Reporter Assay kit (Promega Corporation).

### Western blot

Total protein was extracted with PIPA buffer with protease inhibitors on ice for 20 min. After quantification of protein concentrations using Bicinchoninic Acid Assay, the proteins (20 μg) were separated to 10% SDS-PAGE followed by transferring to PVDF membranes (Millipore). Next, blocking with 5% non-fat milk for 1 h, the membranes were then cultured with primary antibodies overnight at 4°C, followed by incubating at room temperature for 1 h with a HRP-conjugated goat anti-rabbit secondary antibody (ProteinTech Group, dilution 1:2000, cat. no. SA00001-2). After washing with blocking solution, the membranes were determined by an enhanced chemiluminescence (ECL) system (Immun-Star™ HRP chemiluminescent detection kit, Bio-Rad Laboratories). β-Actin as an internal control was used in the present study. The semi-quantification of proteins was carried out using ImageJ software. The primary antibodies (dilution 1:1000, Cell Signaling Technology) include MDM2 (cat. no. 86934), Bcl-2 (cat. no. 3498), p53 (cat. no. 2527), Bax (cat. no. 14796), and β-actin (cat. no. 4970).

### Statistical analysis

All experimental data from three independent experiments were expressed as means ± standard deviations (SD), and analyzed by GraphPad Prism 7 software. Statistical analysis between two groups was calculated by Student’s *t*-test. The comparison among multiple groups was evaluated with one-way ANOVA. A *P*-value<0.05 was considered statistically significant.

## Results

### The effect of LINC00657 on the growth of EC cells

**Figure 1:**
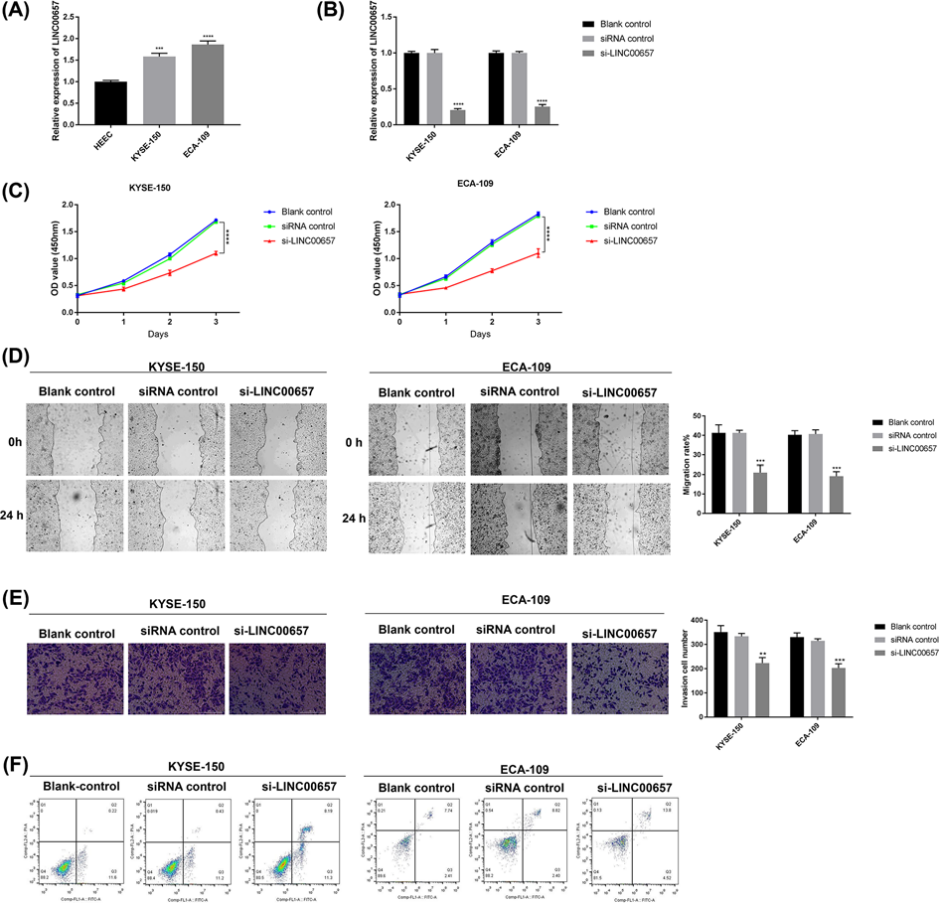
The effect of LINC00657 on the growth of EC cells (**A**) The expression of LINC00657 in EC cells was detected by qRT-PCR. (**B**) The level of LINC00657 in EC cells transfected with si-LINC00657 was measured by qRT-PCR. After EC cells were transfected with si-LINC00657, the (**C**) cell proliferation, (**D**) migration, (**E**) invasion, and (**F**) apoptosis was respectively determined by CCK-8, wound healing assay, transwell assay, and flow cytometry. **P<0.01, ***P<0.001, ^****^P<0.0001 versus siRNA control

To investigate the effect of LINC00657 on EC cells, we detected the level of LINC00657 in EC cells and then determined the proliferation, migration, invasion, and apoptosis of EC cells transfected with si-LINC00657. As shown in Figure 1A, qRT-PCR analysis indicated the aberrant high expression level of LINC00657 in EC cell lines (KYSE-150, ECA-109) as compared with human normal esophageal epithelial cells (HEEC). In addition, LINC00657 siRNA (si-LINC00657) significantly decreased the expression of LINC00657 in EC cells when compared with the blank and siRNA control groups, and thus the transfection of si-LINC00657 was effective (Figure 1B).

The results from CCK-8 assay indicated that EC cell proliferation was obviously inhibited by LINC00657 knockdown (Figure 1C). Besides, wound healing assay and transwell invasion assay revealed that LINC00657 knockdown suppressed the migration and invasion of EC cells (Figure 1D,E). Furthermore, EC cell apoptosis was induced by LINC00657 knockdown in comparison with other control groups (Figure 1F). Therefore, the knockdown of LINC00657 effectively suppressed the proliferation, migration and invasion of EC cells and yet induced cell apoptosis.

### LINC00657 functions as a sponge of miR-26a-5p

**Figure 2:**
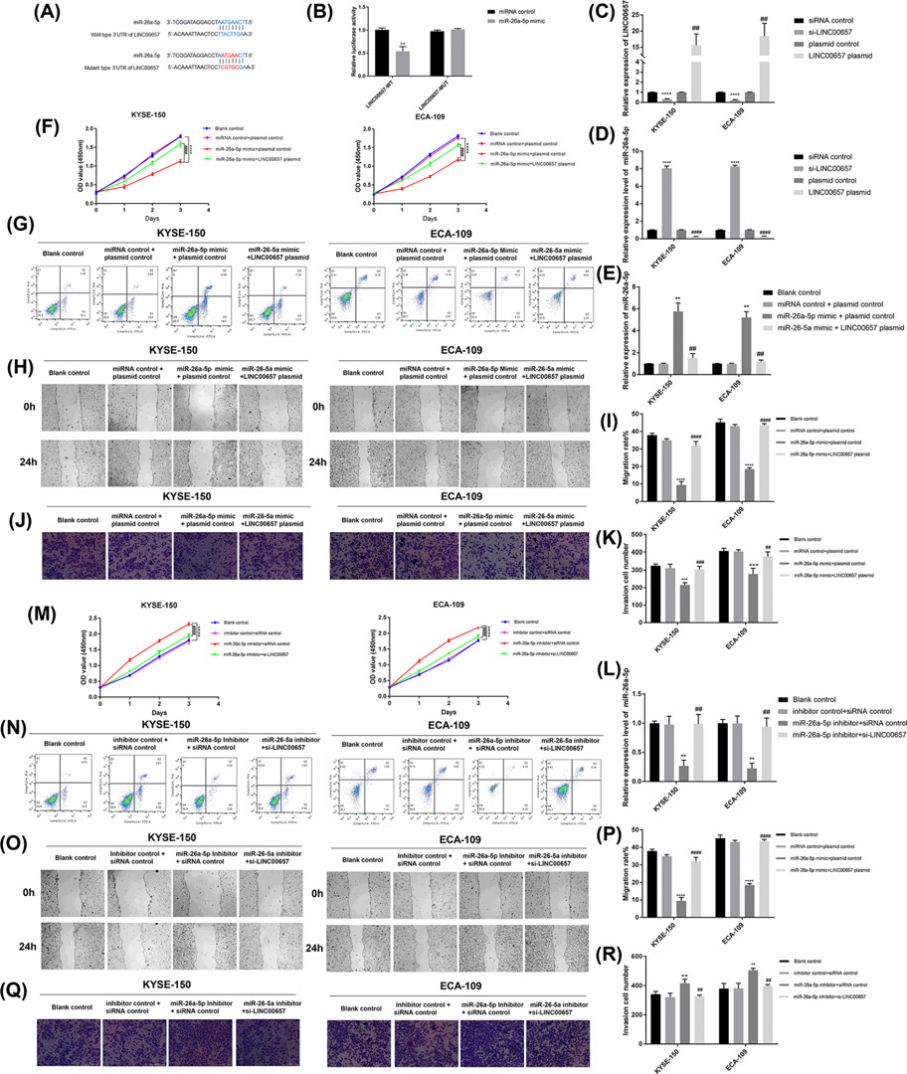
LINC00657 functions as a sponge of miR-26a-5p (**A**) The putative binding sites between LINC00657 and miR-26a-5p were listed. (**B**) The binding relationship between LINC00657 and miR-26a-5p was validated by luciferase reporter assay. (**C** and **D**) After EC cells were transfected with si-LINC00657, LINC00657 plasmid, or control vectors, the expression levels of LINC00657 and miR-26a-5P were examined by qRT-PCR. After miR-26a-5p mimic, or combined with LINC00657 plasmid was transfected into EC cells, (**E**) the expression of miR-26a-5p was measured by qRT-PCR; the (**F**) cell proliferation, (**H** and **I**) migration, (**J** and **K**) invasion and (**G**) apoptosis was respectively detected by CCK-8, wound healing assay, transwell assay, and flow cytometry. After miR-26a-5p inhibitor, or combined with si-LINC00657 was transfected into EC cells, (**L**) the expression of miR-26a-5p was measured by qRT-PCR; the (**M**) cell proliferation, (**O** and **P**) migration, (**Q** and **R**) invasion, and (**N**) apoptosis was respectively determined by CCK-8, wound healing assay, transwell assay, and flow cytometry; **P<0.01, ***P<0.001, ^****^P< 0.0001 versus siRNA control or miRNA control +plasmid control; ^##^*P*<0.01, ^###^*P*<0.001, ^####^*P*<0.0001 versus miR-26a-5p mimic + plasmid control.

Based on ‘ceRNA hypothesis’, we applied starBase website (http://starbase.sysu.edu.cn/index.php) to identify the potential miRNAs binding to LINC00657. As miR-26a-5p has been found to be a suppressor of EC [26], we selected miR-26a-5p as a candidate in the present study. The putative binding sites of LINC00657 with miR-26a-5p were shown in Figure 2A. The relative luciferase activity in EC cells was greatly decreased by the co-transfection of miR-26a-5p mimic and LINC00657-WT vector, while the activity had no obvious change in other groups. Thus, the direct binding relationship of LINC00657 with miR-26a-5p was further validated via luciferase reporter assay (Figure 2B). Besides, there was a high transfection efficiency of si-LINC00657 and LINC00657 plasmid in EC cells (Figure 2C) Moreover, the expression of miR-26a-5p was enhanced after EC cells transfected with si-LINC00657, and miR-26a-5p level was down-regulated by the transfection of LINC00657 plasmid (Figure 2D). Hence, LINC00657 directly bound with LINC00657, and negatively regulated the level of miR-26a-5p.

To further study the effect of LINC00657 with miR-26a-5p in EC cells, we determined the change of the cellular proliferation, migration, invasion, and apoptosis. RT-qPCR showed that the high level of miR-26a-5p induced by miR-26a-5p mimic was down-regulated by the introduction of LINC00657 plasmid (Figure 2E). The experimental results revealed that the proliferation (Figure 2F), migration (Figure 2H,I), and invasion (Figure 2J,K) of EC cells was inhibited by miR-26a-5p mimic, which was obviously reversed by the introduction of LINC00657 plasmid. The increase of cell apoptosis induced by miR-26a-5p mimic was changed by the introduction of LINC00657 plasmid (Figure 2G).

In addition, RT-qPCR suggested that miR-26a-5p inhibitor suppressed the expression level of miR-26a-5p, which was reversed by the introduction of si-LINC00657 (Figure 2L). miR-26a-5p inhibitor considerably promoted the proliferation (Figure 2M), migration (Figure 2O,P), and invasion (Figure 2Q,R) of EC cells, which was changed after EC cells co-transfected with si-LINC00657 and miR-26a-5p inhibitor. Meanwhile, miR-26a-5p inhibitor decreased the apoptosis of EC cells, but the situation was reversed by the introduction of si-LINC00657 (Figure 2N). Thus, LINC00657 could negatively mediate miR-26a-5p to regulate the proliferation, migration, invasion, and apoptosis of EC cells.

### CKS2 is a target of miR-26a-5p

**Figure 3:**
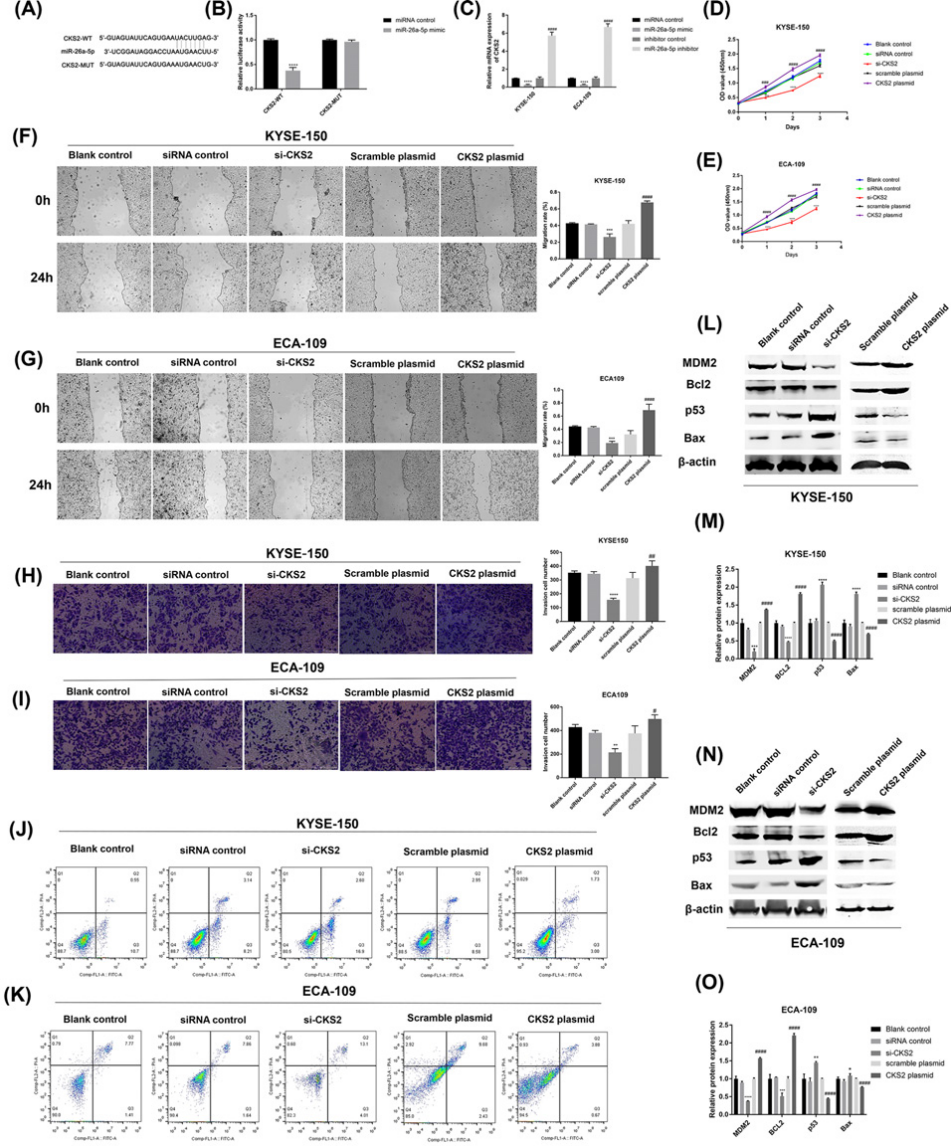
CKS2 is a target of miR-26a-5p (**A**) The putative binding sites between CKS2 and miR-26a-5p were shown. (**B**) Luciferase reporter assay was applied to detect the binding relationship between CKS2 and miR-26a-5p. (**C**) The expression of CKS2 in EC cells transfected with miR-26a-5p mimic or miR-26a-5p inhibitor was assessed by qRT-PCR. After EC cells transfected with si-CKS2 or CKS2 plasmid, the (**D** and **E**) cell proliferation, (**F** and **G**) migration, (**H** and **I**) invasion, and (**J** and **K**) apoptosis was respectively determined by CCK-8, wound healing assay, transwell assay, and flow cytometry. (**L–O**) The protein expression of MDM2, Bcl2, p53, and Bax was evaluated using Western blot after EC cells transfected with si-CKS2 or CKS2 plasmid. **P<0.01, ***P<0.001, ^****^P<0.0001 versus siRNA control or miRNA control +plasmid control; ^####^*P*<0.0001 versus inhibitor control

TargetScan software (http://www.targetscan.org/vert_72/) was utilized to identify the potential gene target of miR-26a-5p. As the high expression of CKS2 (cyclin-dependent kinase subunit 2) is positively correlated with the poor prognosis of EC [23], CKS2 was chosen as a candidate for the further analysis. The binding sites of CKS2 with miR-26a-5p were listed in Figure 3A, which was further validated by luciferase reporter assay (Figure 3B). Moreover, qRT-PCR indicated that the transfection of miR-26a-5p mimic in EC cells decreased the expression of CKS2, and yet miR-26a-5p inhibitor increased CKS2 expression in EC cells (Figure 3C). Thus, miR-26a-5p negatively targeted CKS2.

In addition, we determined the effect of CKS2 on EC cells using CCK-8, wound healing assay, transwell assay, and flow cytometry. The results suggested that CKS2 knockdown considerably suppressed the proliferation (Figure 3D,E), migration (Figure 3F,G), and invasion (Figure 3H,I) of EC cells, and yet induced apoptosis (Figure 3J,K). However, CKS2 overexpression promoted the proliferation, migration, and invasion of EC cells (Figure 3D–I) but repressed apoptosis (Figure 3J,K).

Furthermore, we detected the effect of CKS2 on MDM2/p53/Bcl2/Bax pathway that is critical for the growth of cells. Western blot revealed that the knockdown of CKS2 down-regulated the level of MDM2 and Bcl-2 but up-regulated the level of p53 and Bax (Figure 3L–O). CKS2 overexpression increased the expression of MDM2 and Bcl-2 and yet decreased the expression of p53 and Bax (Figure 3L–O). Collectively, CKS2 could regulate the proliferation, migration, invasion, and apoptosis of EC cells through mediating the MDM2/p53/Bcl2/Bax pathway.

### LINC00657 regulates the growth of EC cells through MDM2/p53/Bcl2/Bax pathway

**Figure 4:**
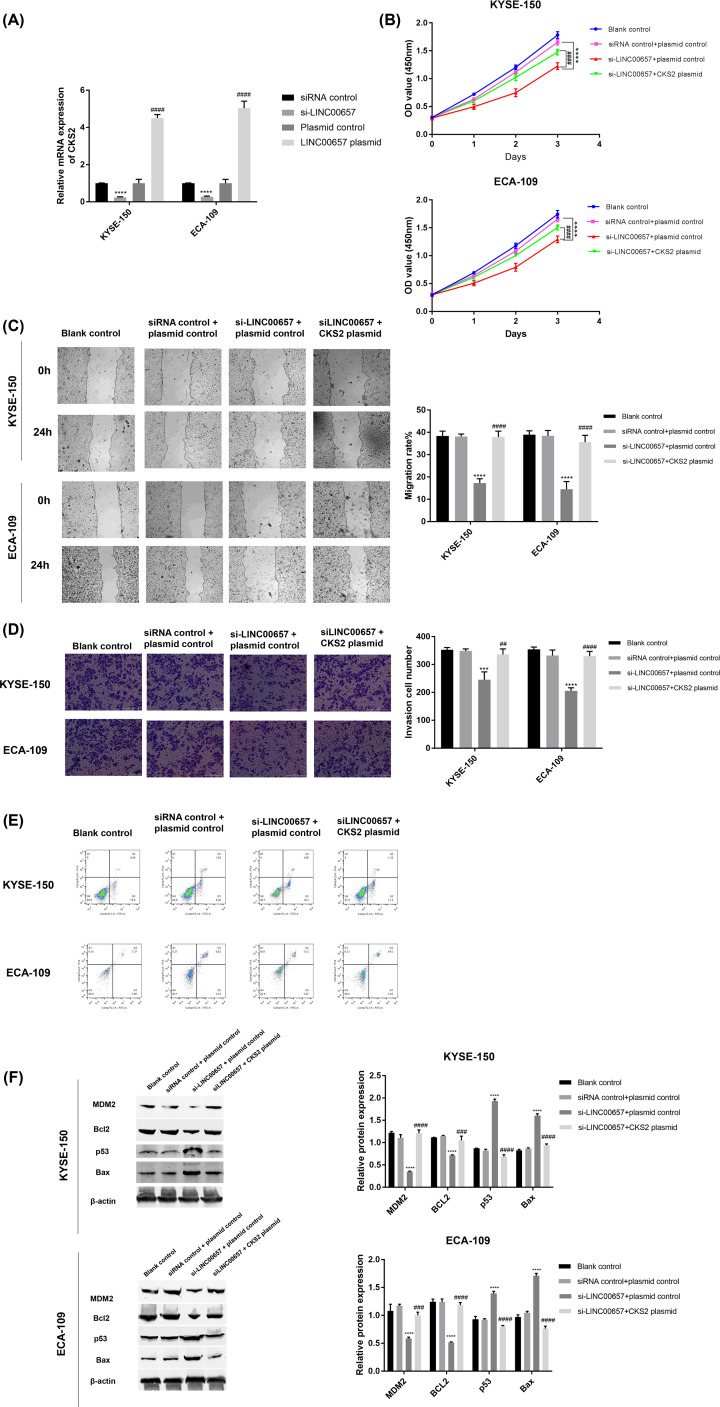
LINC00657 regulates the growth of EC cells through MDM2/p53/Bcl2/Bax pathway (**A**) After EC cells transfected with si-LINC00657, LINC00657 plasmid, or control vectors, the mRNA expression of CKS2 was assessed by qRT-PCR. After si-LINC00657, or combined with CKS2 plasmid was transfected into EC cells, the (**B**) cell proliferation, (**C**) migration, (**D**) invasion, and (**E**) apoptosis was respectively determined by CCK-8, wound healing assay, transwell assay and flow cytometry. (**F**) The protein expression of MDM2, Bcl2, p53, and Bax was determined using Western blot. ***P<0.001, ^****^P<0.0001 versus siRNA control or siRNA control + plasmid control or miRNA control +plasmid control; ^##^*P*<0.01, ^###^*P* <0.001, ^####^*P*<0.0001 versus plasmid control or si-LINC00657 + plasmid control

According to the above experimental results, we speculated that there was a positive correlation of LINC00657 with CKS2. The qRT-PCR analysis showed that LINC00657 knockdown decreased the level of CKS2 and yet LINC00657 overexpression increased CKS2 expression, which further confirmed the positive correlation of LINC00657 with CKS2 (Figure 4A).

In addition, the inhibition of EC cell proliferation, migration, and invasion induced by LINC00657 knockdown was reversed after the cells co-transfected with si-LINC00657 and CKS2 plasmid (Figure 4B–D). LINC00657 knockdown promoted the apoptosis of EC cells, which was changed by the introduction of CKS2 plasmid (Figure 4E). Moreover, the decrease of MDM2 and Bcl-2 and the increase of p53 and Bax induced by LINC00657 knockdown were reversed by the introduction of CKS2 plasmid (Figure 4F). Therefore, LINC00657 mediated CKS2 to regulate the growth of EC cells through MDM2/p53/Bcl2/Bax pathway.

## Discussion

In the present study, LINC00657 was observed to be overexpressed in EC cells (KYSE-150, ECA-109) as compared with normal esophageal epithelial cells (HEEC), which promoted the growth of EC cells. Additionally, the results showed that LINC00657 functioned as a sponge of miR-26a-5p, and CKS2 was identified to be a target of miR-26a-5p. Meanwhile, the anticarcinogenic effect of miR-26a-5p and the oncogenic roles of CKS2 were both validated in EC. Moreover, our study proved the positive correlation between LINC00657 and CKS2, and the knockdown of LINC00657 inhibited CKS2 expression to suppress the proliferation, migration, and invasion of EC cells and induced apoptosis through regulating the MDM2/p53/Bcl2/Bax pathway. Collectively, LINC00657 could mediate miR-26a-5p/CKS2 axis to promote the progression of EC.

To date there are no therapeutic options to effectively improve EC patient’s overall survival [8]. Exploring the molecular mechanism of EC can facilitate the development of EC therapeutic method. LncRNAs have attracted lots of attention because of their key regulatory role in various cancers [11]. LINC00657 as a new identified lncRNA has been reported to function as an oncogenic factor or tumor suppressor in different cancers [13–16]. Although LINC00657 has been found to be overexpressed in ESCC and correlates with patient’s poor prognosis [17], the detailed molecular mechanism of LINC00657 in EC is still unclear. To explore this issue, our study observed the overexpression of LINC00657 in EC cells. The knockdown of LINC00657 suppressed the EC cell proliferation, migration, and invasion, but induced apoptosis. Hence, LINC00657 could be considered as a potential biomarker for the therapy and diagnosis of EC.

ceRNA (competitive endogenous RNA) hypothesis suggests that lncRNAs act as sponges of miRNAs to regulate the target gene expression [27]. In addition, lncRNA–miRNA–mRNA ceRNA networks are relevant with the tumorigenesis and progression [28]. Thus, we used starBase and TargetScan websites to find miR-26a-5p binding to LINC00657 and obtain CKS2 as a target of miR-26a-5p. Previous studies have indicated that miR-26a-5p as a tumor suppressor can suppress the cellular growth but induce apoptosis in diverse cancers [29–31]. Besides, miR-26a-5p has shown an aberrant low expression in ESCC, which promotes cell G1 phase arrest and growth inhibition [19]. In agreement with previous reports, our study found miR-26a-5p functioned as a tumor suppressor in EC cells. Additionally, LINC00657 negatively mediated miR-26a-5p to regulate the growth of EC cells. Furthermore, CKS2 was identified to be a target of miR-26a-5p, and its expression was negatively regulated by miR-26a-5p. The aberrantly high expression of CKS2 observed in in different cancers including EC was correlated with patient’s poor prognosis [22,23]. Consistent with these finding, the present study revealed that CKS2 promoted the growth of EC cells. In addition, CKS2 is involved in the pathogenesis of different cancers through controlling the expression of many molecules such as caspase-3, p53, and Bax [32–34]. Our work also found that CKS2 knockdown significantly decreased the level of MDM2 and Bcl-2 but increased the level of p53 and Bax, whereas CKS2 overexpression up-regulated the expression of MDM2 and Bcl-2 and yet down-regulated the expression of p53 and Bax.

The positive correlation of LINC00657 with CKS2 was proved in the present study. Moreover, LINC00657 could up-regulate CKS2 to increase the level of MDM2 and Bcl-2 but decrease the level of p53 and Bax. p53 is known as a tumor suppressor, and associated with proliferation, apoptosis and cell cycle arrest [35]. MDM2 (murine double minute 2) is an upstream regulator of p53, and its overexpression can lead to the inactivation of p53, thereby facilitating tumorigenic processes [36]. Bcl-2 antagonizes the roles of p53 through inhibiting cell death [37]. Bax as a pro-apoptotic protein is critical for cell apoptosis [38,39]. Moreover, in response to different cellular stresses, p53 can regulate apoptosis via a transcription-dependent and transcription-independent manner [40]. In the transcription-dependent manner, p53 stability is tightly mediated by MDM2, and MDM2 binds to the transactivation domain of p53 (p53TAD) to suppress the p53 transactivation activity and induce p53 proteasomal degradation [41]. MDM2 overexpression in many cancers impairs p53-mediated tumor-suppressor functions, leading to tumor growth [42]. In the transcription-independent manner, p53 in the mitochondria interacts with anti-apoptotic proteins Bcl-2 and Bcl-X_L_, then releasing pro-apoptotic proteins Bax and Bak, thereby triggering apoptosis [40]. Our study observed the aberrantly high expression of MDM2 in EC cells decreased the level of p53, which caused the increase of Bcl-2 and the decrease of Bax, resulting in the inhibition of EC cell apoptosis. Therefore, these findings suggested that LINC00657 mediated miR-26a-5p/CKS2 axis to promote the proliferation, migration and invasion of EC cells but suppress apoptosis through regulating the MDM2/p53/Bcl2/Bax pathway.

In conclusion, LINC00657/miR-26a-5p/CKS2 ceRNA network promoted the growth of EC cells through regulating the MDM2/p53/Bcl2/Bax pathway. These findings are good for understanding the molecular mechanism of EC and provide the potential biomarkers for the therapy and diagnosis of EC.

## Abbreviations

BE: Barrett’s esophagus
CCK-8: cell counting kit-8
ceRNA: competitive endogenous RNA
EAC: esophageal adenocarcinoma
EC: esophageal cancer
ESCC: esophageal squamous cell carcinoma
